# Pulmonary Mucormycosis and hydatid cyst: A case report

**DOI:** 10.1002/ccr3.6496

**Published:** 2022-11-05

**Authors:** Samira Akbarieh, Behrooz Naghili, Hamed Valizade, Samad Beheshtirouy, Behnam Sajedi, Morteza Haramshahi

**Affiliations:** ^1^ Tabriz University of Medical Sciences Tabriz Iran

**Keywords:** case report, cyst hydatid, lung infection, Mucormycosis

## Abstract

Mucormycosis is a group of life‐threatening diseases caused by a fungus of the Mucoraceae family and has a higher mortality rate compared with other known fungal infections. Hydatid cyst, caused by Echinococcus, is a crucial health concern in endemic areas and the disease is characterized by slow‐growing cysts in the liver, lungs, or other organs. In this report, a woman with coexistence of hydatid cyst and Mucormycosis is introduced. The patient was a 52‐year‐old woman with approximately 6 years' history of uncontrolled diabetes mellitus and hypothyroidism, who presented with cough, sputum, and dyspnea 2 months ago. On the initial auscultation of the lungs, there was a decreased sound at the base of the left lung, and she had a fever. In blood tests, she had a high titer of erythrocyte sedimentation rate and 3+ C‐Reactive Protein. The symptoms in favor of hydatid cyst were observed in lung computed tomography and in pleural needle biopsy, hydatid cyst was confirmed. With this indication, she underwent wedge resection, and resection of the left lower lung cyst. Two samples are taken from the cyst side and the pathology report was consistent with Mucormycosis (wide filaments with a 90‐degree angle). The patient was immediately treated with liposomal amphotericin for 4 weeks. The Lung CT scan was performed before and after treatment. Albendazole was treated to treat hydatid cyst. After discharge, the treatment of the patient continued with oral Posaconazole, and after the treatment finalization, the general condition of the patient was good, and she did not have any complaints. In pulmonary diseases that do not respond significantly to surgical treatment (such as hydatid cyst), fungal disease (mucor) must be considered simultaneously. Mucormycosis is more prevalent in patients with uncontrolled diabetes, and it is necessary to be considered if these patients were infected with pneumonia and their symptoms did not improve with usual treatments.

## Graphical abstract

In this case report, we present a patient with uncontrolled diabetes with pneumonia symptoms treated with different antibiotics which did not improve the patient's state. After diagnosis of lung hydatid cyst by needle biopsy. During the surgery for hydatid cyst, based on the possibility of a simultaneous fungal and bacterial infection, a sample was taken from the side cavity. Resection was performed, and Mucormycosis was diagnosed which was confirmed by two pathologists and patient underwent pharmacotherapy. The patient completely recovered which is very rare in such setting.

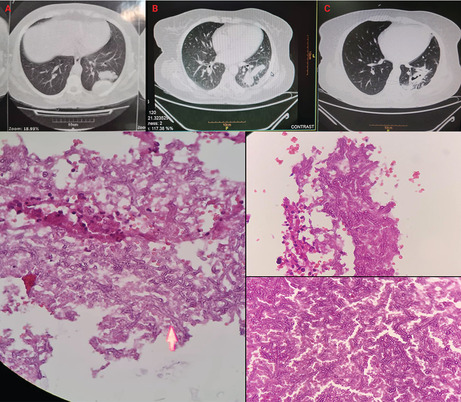

## 1 INTRODUCTION

Mucormycosis is an uncommon and rapidly progressive invasive fungal infection that can in case of lack of timely diagnosis and treatment, lead to mortality. It is usually caused by members of the Mucoraceae family of fungi, which subgroups include Rhizopus, Mucor, and Absidis. Mucormycosis is transmitted through the air.^1^ This fungus is not normally pathogenic and only occurs when the patient has underlying diseases such as immunodeficiency and diabetic ketoacidosis.^2^ Other conditions of immunodeficiency and the use of drugs that weaken the immune system are the other predisposing factors.

Pulmonary Mucormycosis is the second most common manifestation of mucositis, after the Rhinocerebral form. The symptoms of this infection include dyspnea, cough, chest pain, and hemoptysis. Vascular invasion causes necrosis, cavitation, or hemoptysis. Lobar opacity, solitary mass, nodular disease, cavity, or wedge‐shaped infarcts may be seen on chest X‐rays.^3^, ^4^ The diagnosis of Mucormycosis is a medical challenge and autopsy studies have shown that up to half of cases were diagnosed only after death.^5^

Hydatid cyst, caused by Echinococcus, is a cestode parasite of the tapeworm variant. Humans are mostly affected by two species: granulosus and multilocularis which cause two major types of the disease; cystic echinococcosis and alveolar echinococcosis.^6^ Hydatid cyst is a crucial health problem in endemic areas and the disease is characterized by slow‐growing cysts.

The lungs are the second most prevalent site of this infection after the liver. Manifestations of pulmonary hydatid cyst vary depending on whether the cyst is intact or ripped and their clinical manifestations mostly depend on size and location. Unripped cysts have no specific symptoms. Large cysts may cause signs of dry cough, pressure on adjacent organs, and upper superior vena cava syndrome. Also, atelectasis may be seen due to the compressive effect on the bronchi. Diagnosis of a cystic mass in a person who has a history of contact with dogs and lives in endemic areas strongly suggests a hydatid cyst.^7^, ^8^ The treatment method for hydatid cyst of the lung is surgery and drug treatments are used in cases where surgery is not possible for any reason.^9^

The coexistence of pulmonary Mucormycosis and hydatid cyst is a rare phenomenon and best of our knowledge this is the first‐ever reported case of coexistence of these two pathogens. Here, we report the symptoms, diagnosis, treatment, and follow‐up details of our presented case of coexistence of pulmonary Mucormycosis and hydatid cyst in Imam Reza Medical Research and Training hospital of The Tabriz University of Medical Science.

## 2 CASE PRESENTATION

A 52‐years‐old woman with a history of uncontrolled diabetes, an HbA1C level of 12%, and hypothyroidism for about 6 years, was referred to the hospital due to productive cough, hemoptysis, fever, and chills started two months before, and it was accompanied by shortness of breath. The drug history was metformin and oral Glibenclamide. At the time of admission, she had a fever of 38° centigrade, a 95% oxygen saturation level, a respiratory rate of 23, and a heart rate of 90. In the physical examination, she was pale; the cardiac auscultation was normal and, in the skin examination, the scars of the abdomen were evident. On the initial auscultation of the lungs, there was a decreased sound at the base of the left lung, and she had a fever. In blood tests, she had a high titer of erythrocyte sedimentation rate (ESR) and 3+ C‐Reactive Protein (CRP). We were during SARS Coronavirus 2 PCR test was conducted and it was negative. The case has no history of steroid intake and HIV was ruled out.

**FIGURE 1:**
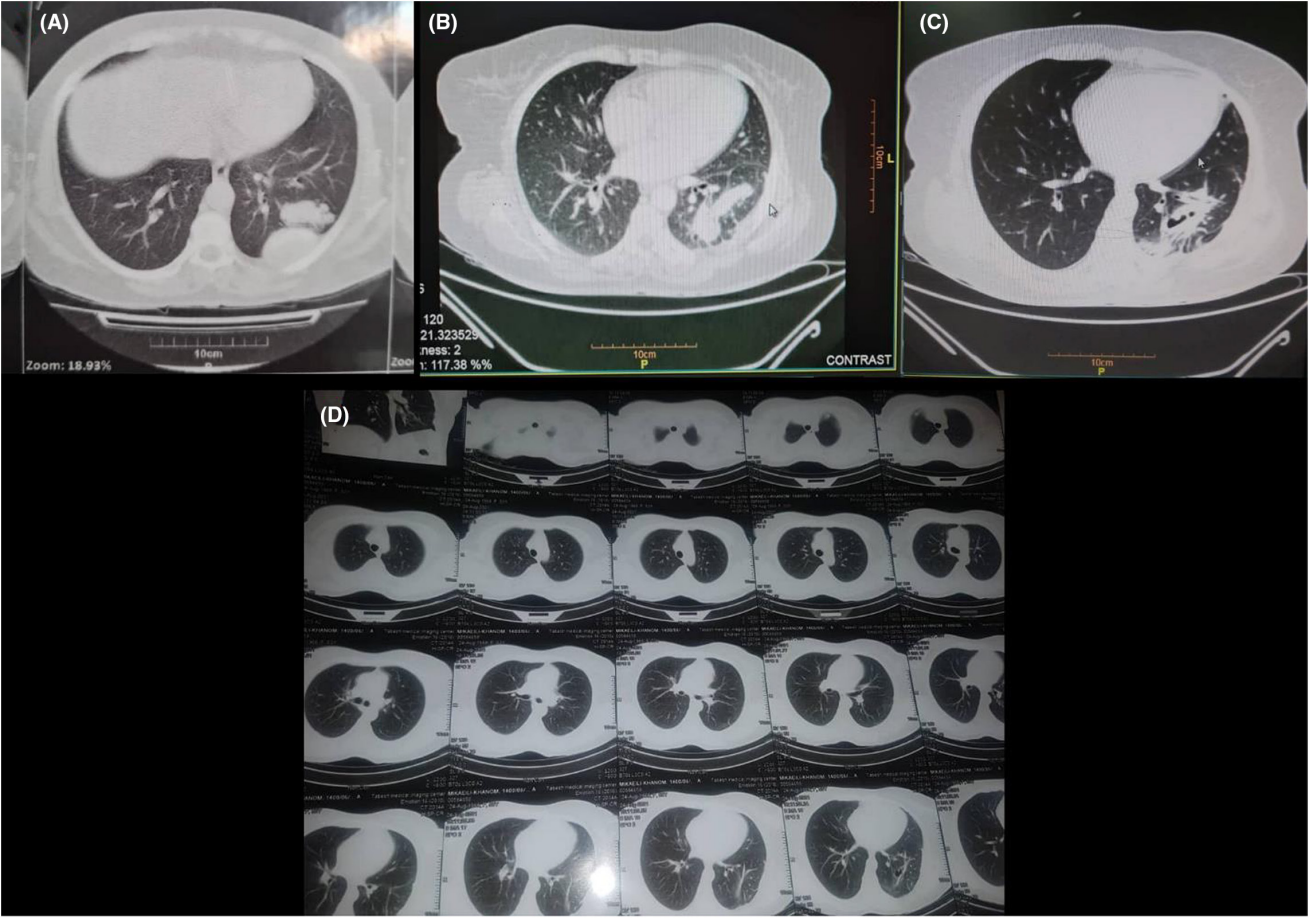
CT scans (A: before surgery; B: After surgery; C: After surgery and amphotericin B therapy; D: 6 months after the treatment and follow‐up).

Due to pulmonary symptoms, she was repeatedly treated with antibiotics during this period which did not have a clinical response. In our hospital, after observing the findings in favor of hydatid cyst in lung computed tomography (CT‐scan) (Figure 1A), the patient underwent a biopsy, and after reporting a positive serology for hydatid cyst, medical treatment with two albendazole tablets, once daily, was initiated. The patient's symptoms continued until she underwent a thoracotomy and resection of the left lower lung cyst. During the surgery, a sample was taken from another cavity located next to the resected cyst (Figure 1B).

**FIGURE 2:**
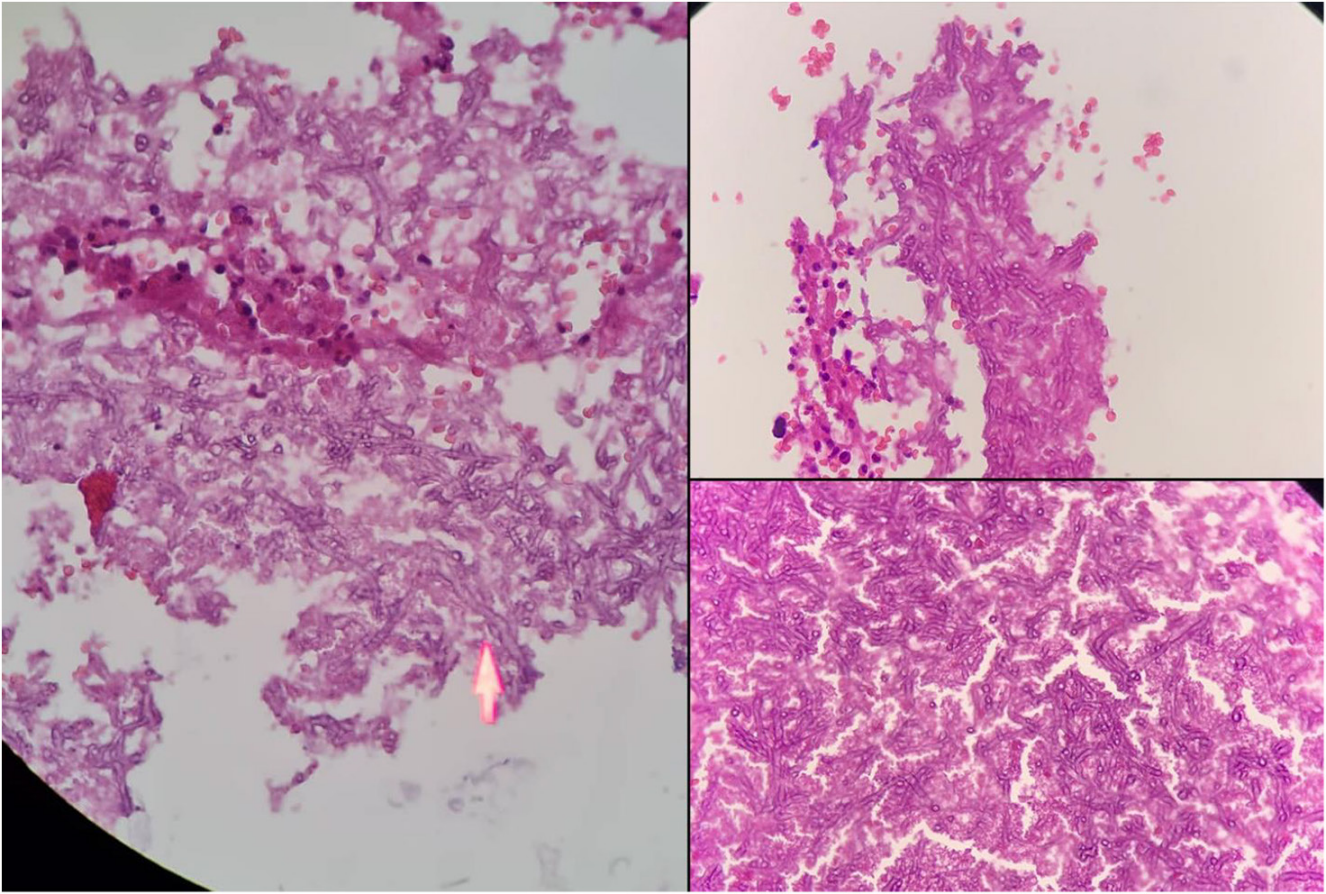
Pathology slides of the patient. The marker shows the Mucormycosis.

In pathology report in left chest wall lesion resection, “consist with hydatid cyst accompanied by foreign body reaction” and in the left lung, lower lesion resection, “lung tissue with hemorrhage, congestion, infiltration cells with neutrophil predominance and groups of large, nonseptic hyphae with variable width, 90‐degree angle branching and non‐parallel walls consistent with Mucormycosis” were noted (Figure 2).

After surgery and confirmation of the coexistence of lung hydatid cyst and Mucormycosis, the patient was treated with liposomal amphotericin B 5 mg/kg = 300 mg daily for 4 weeks and hydatid cyst medical therapy also continued with albendazole 80 mg daily. After 4 weeks of treatment with amphotericin B, a lung CT scan reported an improvement in the condition (Figure 1C). During the four weeks treatment period, the patient's symptoms were stable, and she did not have fever and chills and did not report any complaints about the previous symptoms. Liposomal amphotericin b side effects were not reported to our patient upon request for a daily check.

The oral treatment continued, and the patient was discharged after 2 weeks in good general condition. For maintenance therapy, the patient received itraconazole 200 mg tablets, BID to complete 6 weeks of treatment. At the end of 6 months of follow‐up, she had no pulmonary complaints and clear radiological evidence was noted in her lung CT scan. (Figure 1D) and after two weeks, she was revisited without any particular complaints.

## 3 DISCUSSION

Here, we reported a poorly controlled patient with diabetes mellitus and coexistence of lung Mucormycosis and hydatid cyst, which she was treated successfully with medical and surgical treatments. Based on imaging evidence and clinical findings, finally, the appropriate therapeutic response to treatment was observed. Best of our knowledge, this is the first reported case of coexistence of pulmonary Mucormycosis and hydatid cyst.

In our reported case, the patient was taken to the operating room with a diagnosis of hydatid cyst, which is an indication of thoracotomy, and the pathology finding was consistent with Mucormycosis. The most accurate diagnostic method for fungi infections is to perform a biopsy and observe vascular invasion and subsequent tissue thrombosis with evidence of acute and chronic inflammation in the sample. In our case, the surgical biopsy was sent for pathologic assessments and 90‐degree angle branching and non‐parallel walls consistent with Mucormycosis were reported.

In patients with symptoms of pneumonia who do not respond to the treatment, Mucormycosis should be considered from a diagnostic perspective. The presence of this infection and the lack of a standard protocol for treatment is experimental for now.^10^ In general, dealing with patients with Mucormycosis includes natural treatment and elimination of the underlying factor. Because of underlying conditions, in the present case, amphotericin b consumption was started.

The prognosis of Mucormycosis depends on several factors, including rapid diagnosis recovery, and the underlying factor. Studies in this regard have also yielded favorable results from the administration of hyperbaric oxygen and liposomal nystatin. Liposomal amphotericin is the most effective drug known for the medical treatment of Mucormycosis. The duration and dose of the drug depend on various factors, including the side effects, patient tolerance, and the progress of the disease.^11^ The Survival rate is higher using the combination of medical and surgical treatment.^12^ Our patient was treated with liposomal amphotericin B and itraconazole 200 mg as maintenance therapy. According to the patient's improvement in the treatment process and also other studies in this field, it turns out that it was an appropriate treatment.^13^

## 4 CONCLUSION

Mucormycosis and hydatid cyst can be presented together. In diabetic patients or other immunosuppressive conditions with symptoms of pneumonia who do not respond appropriately to the usual treatments, Mucormycosis should be considered. In Mucormycosis patients with underlying conditions such as diabetes mellitus, quick diagnosis and initiation of the treatment improve the survival rate of the patients.

## AUTHOR CONTRIBUTIONS

Samira Akbarieh, Behrooz Naghili, Hamed Valizade, Samad Beheshtirouy, Behnam Sajedi, and Morteza Haramshahi were on the treatment team of the patients. Behnam Sajedi and Samira Akbarieh drafted the manuscript and Behrooz Naghili, Hamed Valizade, Samad Beheshtirouy, and Morteza Haramshahi critically revised it. Samira Akbarieh, Hamed Valizade, and Behnam Sajedi prepared the figures. All authors read and approved the final manuscript.

## FUNDING INFORMATION

None.

## CONFLICT OF INTEREST

None.

## ETHICAL STATEMENT

The patient was informed from this case report and a written consent form was obtained.

## CONSENT

Written informed consent was obtained from the patient for publication of this case report and any accompanying images. A copy of the written consent is available for review by the Editor‐in‐Chief of this journal.

## Data Availability

Not applicable.
